# Are women who quit smoking at high risk of excess weight gain throughout pregnancy?

**DOI:** 10.1186/s12884-016-1056-z

**Published:** 2016-09-06

**Authors:** Adam Hulman, Olha Lutsiv, Christina K. Park, Lynette Krebs, Joseph Beyene, Sarah D. McDonald

**Affiliations:** 1 Department of Obstetrics & Gynecology, McMaster University, 1280 Main Street West, Room 3N52, Hamilton, ON L8S 4K1 Canada; 2 Department of Medical Physics & Informatics, University of Szeged, Szeged, Hungary; 3 Department of Clinical Epidemiology & Biostatistics, McMaster University, Hamilton, ON Canada; 4 Departments of Obstetrics & Gynecology, Radiology, Clinical Epidemiology & Biostatistics, McMaster University, Hamilton, ON Canada

**Keywords:** Pregnancy, Smoking, Gestational weight gain, Piecewise mixed-effects model, Longitudinal analysis

## Abstract

**Background:**

Smoking cessation has been reported to be associated with high total gestational weight gain (GWG), which itself is a risk factor for adverse maternal-infant outcomes. Recent studies have criticized conventional single measures of GWG, since they may lead to biased results. Therefore, we aimed to compare patterns of GWG based on serial antenatal weight measurements between women who: never smoked, quit during pregnancy, continued to smoke.

**Methods:**

Participants (*N* = 509) of our longitudinal study were recruited from seven antenatal clinics in Southwestern Ontario. Serial GWG measurements were abstracted from medical charts, while information on smoking status was obtained from a self-administered questionnaire at a median gestational age of 32 (27–37) weeks. GWG patterns were assessed by fitting piecewise mixed-effects models. First trimester weight gains and weekly rates for the last two trimesters were compared by smoking status.

**Results:**

During the first trimester, women who never smoked and those who quit during pregnancy gained on average 1.7 kg (95 % CI: 1.4–2.1) and 1.2 kg (0.3–2.1), respectively, whereas women who continued smoking gained more than twice as much (3.5 kg, 2.4–4.6). Weekly rate of gain in the second and third trimesters was highest in women who quit smoking (0.60 kg/week, 0.54–0.65), approximately 20 and 50 % higher than in women who never smoked and those who smoked during pregnancy, respectively.

**Conclusions:**

In this longitudinal study to examine GWG by smoking status based on serial GWG measurements, we found that women who quit smoking experienced a rapid rate of gain during the last two trimesters, suggesting that this high-risk group may benefit from targeted interventions.

**Electronic supplementary material:**

The online version of this article (doi:10.1186/s12884-016-1056-z) contains supplementary material, which is available to authorized users.

## Background

Smoking during pregnancy is a major risk factor for adverse perinatal outcomes, including low birthweight, preterm birth, and stillbirth [1]. Interventions promoting smoking cessation have been successful in reducing the proportion of pregnant women who smoke, resulting in some improved pregnancy outcomes among women who quit smoking compared to those who continue [2]. Despite this, in the general population, it is widely recognized that smoking cessation is associated with weight gain, with most of the gain occurring during the first 3 months after cessation [3]. Women who quit smoking before or during pregnancy may thus be at greater risk of excessive gestational weight gain (GWG), but this remains unknown.

High pregnancy weight gain is itself associated with adverse maternal and infant outcomes. High gain doubles women’s risk of becoming overweight after delivery, and becoming obese later in life [4, 5], and also increases the risk of high birth weight, which further doubles the infant’s risk of obesity in childhood and adulthood [6–8]. Recognizing the risks associated with excessive GWG, the Institute of Medicine (IOM) published revised GWG guidelines in 2009 [9]. Despite the existing guidelines, the majority of pregnant women continue to gain above recommendations [10]. Given that GWG is one of the few potentially modifiable risk factors for adverse perinatal outcomes [11–13], examining its determinants is an important step towards improving maternal and neonatal outcomes.

Recently, it has been recognized that conventional measures of GWG, such as total GWG, may introduce substantial bias to studies of GWG, resulting in the stating of a significant association even if none exists [14], since pregnancies of longer duration tend to have higher weight gain. Other clinically relevant issues are that total GWG is known only at the end of the pregnancy when there are no more opportunities for interventions, and that the timing of weight gain is not captured by total GWG [15]. Alternative approaches using serial antenatal GWG measures resolve this bias in conventional measures. Additionally, serial measurements give an understanding of weight gain patterns, which is necessary for developing more effective interventions.

Therefore, the aim of this study was to describe and compare GWG patterns by smoking status using a longitudinal approach based on serial antenatal GWG measurements.

## Methods

### Study setting and population

We conducted a longitudinal study in which the outcome (GWG) was extracted at the end of follow-up from medical charts and the exposure (smoking status) was assessed at a single time point during follow-up using a self-administered survey. Participants were recruited from five obstetric and two midwifery clinics in Southwestern Ontario, Canada, between May and September 2013. Women with viable singleton gestations, without severe morbidities that have an impact on weight gain (e.g., bariatric surgery, bulimia and anorexia), and who could read English sufficiently well to complete the survey were included in the study. The recruitment process and the study protocol is described in full detail elsewhere [16].

### Ascertainment of smoking status and trimester-specific GWG

Our primary exposure of interest—smoking status—was assessed with a self-administered survey during an antenatal visit. Women were asked a series of questions about their smoking habits, which allowed us to categorize them into one of three groups: 1) women who never smoked, 2) women who smoked previously but quit upon finding out that they were pregnant, or 3) women who smoked during pregnancy. Women who quit smoking during pregnancy were those who answered “Yes” to the survey question, “Prior to learning that you were pregnant, did you smoke?”, and subsequently answered “No” to the question, “Now that you’re pregnant, do you smoke?”. Additional information on other maternal pre-pregnancy characteristics, including age, education, household income, marital status, ethnicity, and parity was also obtained from the self-administered antenatal questionnaire.

Women’s medical charts were used to extract information on their pre-pregnancy weight and height, as well as their serial GWG measurements throughout pregnancy. At the participating study clinics, women were weighed by a doctor, nurse or midwife at each of their antenatal visits. Pre-pregnancy weight was self-reported by women and recorded in their medical charts, and if unknown then the first available antenatal weight measurement was used. Gestational age, as recorded in the medical charts, was determined using the best available estimate, which was either early pregnancy ultrasound, or based on the last menstrual period, which was revised by second trimester ultrasound if necessary. Data extraction from medical charts occurred following each woman’s expected date of delivery.

### Statistical analyses

Maternal pre-pregnancy characteristics by smoking status were compared with Fisher’s exact test for categorical variables and with one-way analysis of variance (ANOVA) for continuous variables. GWG trajectories were assessed by fitting mixed-effects models, the recommended method to deal with serial measurements. In order to complement the recommendations in the IOM GWG guidelines [9], we used a piecewise model specification with two linear segments and a breakpoint at the end of the first trimester (13 completed weeks of pregnancy) [17]. This was achieved by including the following terms of time (gestational age) in the model: (1) gestational age itself and (2) gestational age centered at 13 weeks (gestational age—13 weeks), but having zero values until week 13. The second term represented the slope difference between the first period (1st trimester) and the second period (2nd and 3rd trimester). The weekly rate in the 1st trimester was the estimated coefficient of (1), while it was the sum of the coefficients of (1) and (2) for the 2nd and 3rd trimester. Confidence intervals for the latter estimates were calculated using the appropriate model contrasts with the *gmodels* R package. Random effects were included for both (1) and (2). The intercept was dropped from the model to assure that trajectories start at 0 kg at the beginning of the pregnancy. Trajectory differences by smoking status were modeled by including the interaction of smoking status with both (1) and (2) in the model. The model was refitted with two separate adjustments: adjusting only for pre-pregnancy BMI class (underweight: <18.5 kg/m^2^, normal weight: 18.5–25 kg/m^2^, overweight: 25–30 kg/m^2^, obese: ≥30 kg/m^2^), and adjusting for a wider range of covariates, including age, education, household income, marital status, ethnicity, parity and pre-pregnancy BMI class. Piecewise mixed-effects models were fitted with the *nlme* R package. Statistical analyses were performed using R version 3.1.2.

## Results

A total of 585 women were approached in the study clinics, of whom 525 consented to participate in the study. Women were further excluded if they had a fetal demise (*n* = 2), were lost to follow-up (*n* = 8) or had missing information on pre-pregnancy weight (*n* = 1), gestational weight gain (*n* = 3) or smoking status (*n* = 2), which resulted in a final sample of 509 participants, who had complete information on all relevant variables. Participants contributed one (3 %), two (21 %), three (53 %) or four (23 %) serial GWG measurements during pregnancy. Hence, the final sample included 1503 GWG measurements from the 509 participants (Additional file 1: Figure S1). The median (Q1-Q3) gestational age at the time of survey completion, which was used to determine women’s smoking status, was 32 (27–37) weeks. Medians did not differ significantly by smoking status (*P* = 0.44). Neither the proportion of GWG measurements from the 1st trimester (varied between 15 % and 18 %; *P* = 0.74), nor the total number of GWG measurements (Fisher Exact Test: *P* = 0.55) differed significantly by smoking status.

Pre-pregnancy characteristics by smoking status are summarized in Table 1. The majority of women never smoked (80 %), while 12 % quit upon finding out that they were pregnant and 8 % continued to smoke during pregnancy. The cohort was predominantly Caucasian (80 %), which did not differ between the three groups. Women who continued smoking during pregnancy were younger, and a greater proportion of them was less educated, had a lower income, lived alone (single, widowed or divorced), and were obese, compared to women who quit and especially compared to those who never smoked.

**Table 1:** Pre-pregnancy characteristics of the study participants by smoking status

Maternal characteristics	Women who never smoked (*N* = 405)	Women who quit smoking (*N* = 61)	Women who continued to smoke (*N* = 43)	*P*-value^a^
Age (years), mean (SD)	31.3 (5.0)	28.8 (6.3)	27.0 (4.5)	< .001
Education (%)				< .001
High school or less	8.4	27.9	48.8	
Community college or some university	43.2	54.1	41.9	
At least bachelor’s degree	48.4	18.0	9.3	
Household income (%)				< .001
Less than $20,000	5.9	16.4	25.6	
$20,000–$80,000	33.9	49.2	34.9	
Over $80,000	47.8	24.6	16.3	
Preferred not to answer	12.4	9.8	23.3	
Married, common-law or living with partner (%)	93.8	80.3	65.1	< .001
Caucasian (%)	78.2	88.5	85.7	.12
Nulliparous (%)	45.0	61.7	31.7	.01
BMI (kg/m^2^), mean (SD)	25.7 (5.5)	25.5 (6.6)	27.0 (7.2)	.26
BMI class (%)				.03
Underweight (<18.5 kg/m^2^)	3.0	1.6	7.0	
Normal (18.5–24.9 kg/m^2^)	51.6	62.3	44.2	
Overweight (25.0–29.9 kg/m^2^)	26.4	24.6	14.0	
Obese (≥30.0 kg/m^2^)	19.0	11.5	34.9	

*Abbreviation(s)*: *BMI* body mass index

^a^ *P* values were calculated with ANOVA for continuous variables and with Fisher’s exact test for categorical variables

GWG patterns characterized by the total 1st trimester gain (at 13 completed weeks) and weekly rates during the 2nd and 3rd trimester are summarized in Table 2. Women who never smoked and those who quit gained a similar amount (*P* = 0.31) in the 1st trimester: 1.7 kg (95 % CI: 1.4, 2.1) and 1.2 kg (95 % CI: 0.3, 2.1), respectively, while those who smoked during pregnancy gained twice as much (3.5 kg, 95 % CI: 2.4, 4.6).

**Table 2:** Gestational weight gain patterns by smoking status

	Women who never smoked (*N* = 405/ 1,205^a^)	Women who quit smoking (*N* = 61/ 179^a^)	Women who continued to smoke (*N* = 43/ 119^a^)
Unadjusted			
1st trimester			
Total trimester weight gain, kg, (95 % CI)	1.7 (1.4, 2.1)	1.2 (0.3, 2.1)	3.5 (2.4, 4.6)
Mean difference, kg (95 % CI)	Reference	−0.5 (−1.5, 0.5)	1.8 (0.6, 3.0)
2nd and 3rd trimester			
Rates of weight gain, kg/week, (95 % CI)	0.49 (0.47, 0.51)	0.60 (0.54, 0.65)	0.40 (0.32, 0.45)
Mean difference, kg/week (95 % CI)	Reference	0.11 (0.05, 0.17)	−0.10 (−0.17,−0.03)
Adjusted^b^			
1st trimester			
Total trimester weight gain, kg, (95 % CI)	2.3 (1.5, 3.2)	1.7 (0.5, 2.8)	4.7 (3.2, 6.2)
Mean difference, kg (95 % CI)	Reference	−0.7 (−1.7, 0.4)	2.4 (1.1, 3.7)
2nd and 3rd trimester			
Rates of weight gain, kg/week, (95 % CI)	0.51 (0.46, 0.55)	0.59 (0.53, 0.66)	0.42 (0.34, 0.50)
Mean difference, kg/week (95 % CI)	Reference	0.09 (0.03, 0.15)	−0.09 (−0.16,−0.01)

*Abbreviation(s)*: *CI* confidence interval

^a^N represents the number of participants/measurements

^b^Estimates are for a 30-year old, Caucasian, nulliparous, married woman with some college or university education, a household income between $20,000 and $80,000, and a normal BMI

Women who quit smoking gained weight the most rapidly in the 2nd and 3rd trimesters, at a rate of 0.60 kg/week (95 % CI: 0.54, 0.65), which is 22 % (95 % CI: 11, 34) and 53 % (95 % CI: 32, 75) faster than women who never smoked and those who smoked during pregnancy, respectively.

Adjusting for age, education, household income, marital status, ethnicity, parity and pre-pregnancy BMI class modestly increased the differences in 1st trimester total GWG, and slightly decreased the differences in weekly rates. Statistical significance and direction of differences by smoking status did not change when taking these factors into account.

**Fig. 1:**
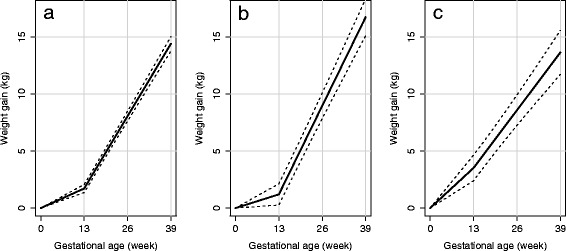
Mean gestational weight gain trajectories assessed with piecewise–linear mixed-effects models in **a** women who never smoked, **b** women who quit smoking, and **c** women who smoked during pregnancy

Mean GWG trajectories with 95 % confidence intervals are displayed in Fig. 1. The different rates of weight gain in the 1st trimester versus the 2nd and 3rd trimesters is clearly apparent among women who never smoked and those who quit, however it is not as obvious in women who continued to smoke during pregnancy, despite a significant slope difference (*P* = .04) between the two time periods. Despite these significantly different patterns of GWG in women who never smoked and those who smoked during pregnancy, both groups would have gained on average approximately 14 kg (*P*-value for difference = .46) by the end of the 39th week, the median gestational age at birth in each group. In contrast, women who quit would have gained on average 16.7 kg (95 % CI: 15.1, 18.4) in the same period.

Weight gain characteristics from the BMI adjusted model are summarized in Table 3. Women who smoked during pregnancy gained more in the 1st trimester than the IOM recommendation of 0.5–2.0 kg, regardless of BMI class. With the exception of obese women who quit smoking, who gained less than the recommended range, all other subgroups gained according to the guidelines in the 1st trimester. The majority of women gained above GWG guidelines in the 2nd and 3rd trimesters. Underweight women who never smoked and underweight and normal weight women who smoked during pregnancy were the only groups to gain within the recommended weekly ranges. Overweight women who quit smoking gained on average more than twice as rapidly as their recommended rate, which was the largest relative difference.

**Table 3:** Total weight gain (kg) in the 1st trimester and weekly rate (kg/week) in the 2nd and 3rd trimesters by smoking status and pre-pregnancy BMI class along with IOM recommendations by BMI class

BMI	IOM recommended gain	Smoking status		
		Women who never smoked	Women who quit smoking	Women who continued to smoke
1st trimester (kg)				
Underweight	0.5–2.0	1.7 (−0.2, 3.5)	1.0 (−1.0, 3.1)	**3.7** (1.6, 5.7)
Normal		2.0 (1.5, 2.4)	1.4 (0.4, 2.3)	**4.0** (2.8, 5.2)
Overweight		2.0 (1.3, 2.6)	1.3 (0.2, 2.4)	**4.0** (2.7, 5.2)
Obese		0.7 (−0.1, 1.5)	0.1 (−1.1, 1.3)	**2.7** (1.5, 3.9)
2nd and 3rd trimester (kg/week)				
Underweight	0.44–0.58	0.52 (0.42, 0.62)	**0.62** (0.50, 0.73)	0.44 (0.33, 0.56)
Normal	0.35–0.50	**0.51** (0.49, 0.54)	**0.61** (0.56, 0.67)	0.44 (0.37, 0.50)
Overweight	0.23–0.33	**0.52** (0.48, 0.55)	**0.62** (0.56, 0.68)	**0.44** (0.37, 0.51)
Obese	0.17–0.27	**0.38** (0.33, 0.42)	**0.48** (0.41, 0.54)	**0.30** (0.23, 0.37)

Bold values are above the recommended IOM range

*Abbreviation(s)*: *BMI* body mass index, *IOM* Institute of Medicine

## Discussion

Using a longitudinal approach based on serial measurements of GWG during pregnancy, we found a strong effect of smoking cessation on GWG patterns: women who quit smoking had a more rapid GWG in the 2nd and 3rd trimester compared to women who never smoked, and even more so compared to women who smoked during pregnancy. Although women who never smoked and those who smoked during pregnancy gained a similar amount of weight by the end of the 39th week, their pattern of gain was markedly different. Interestingly, women who smoked gained double the amount of weight in the first trimester relative to the other two groups, but were on a less steep trajectory during the rest of the pregnancy. These results are relevant from both a clinical and a public health perspective in light of previous findings on how timing of weight gain can influence pregnancy outcomes [15].

Beyond total GWG recommendations by pre-pregnancy BMI class, the 2009 IOM guidelines for GWG include optimal weekly rates for the 2nd and 3rd trimesters [9], which avoid bias because they are not dependent on the length of gestational. However, these calculations were simply extrapolations and not based on longitudinal studies [15]. We found that obese women generally gained less rapidly than the other three BMI classes, who gained at similar rates. These results were in line with data from large cohorts summarized in the 2009 guidelines [9]. Despite this, overweight and obese women gained above the recommended weekly rate in the 2nd and 3rd trimesters regardless of smoking status, as did all women who quit, regardless of BMI class.

The majority of previous studies have investigated the effect of smoking habits either on total GWG [18–20] or on proportions of GWG categories (inadequate, adequate, excessive) as defined by the IOM guidelines from 1990 [21–24] or 2009 [25–27]. In contrast to our analyses, most of these studies were based on a single measure of total GWG. In line with our results, previous studies have consistently shown that the mean total GWG is similar among women who never smoked and those who smoked during pregnancy and that women who quit gained 1.2–3.5 kg more than women who never smoked and even slightly more than women who smoked during pregnancy [18, 19, 25].

Previous work using weekly rates of weight gain in the 2nd and 3rd trimesters [27], but lacking information on pre-pregnancy weight, assumed a 1.25 kg gain in the first trimester, which may have led to erroneous estimates of pre-pregnancy BMI class, due to the larger GWG in this period, especially among women who continued smoking (e.g., 3.5 kg in our study). Most other studies that have investigated trimester-specific GWG have been inconclusive in determining the association between total 1st trimester GWG and smoking status [18, 19, 28], although a study by Karachaliou et al. supports our findings of significantly greater GWG in the 1st trimester among smokers [29].

Analyses based on total GWG measures are prone to bias because of their correlation with gestational age [14]. This could pose a problem when investigating the effect of smoking, because smoking is associated with preterm birth and gestational age. Although new methods using serial measurements have been suggested to overcome this problem, only a few studies to date have investigated the effect of smoking cessation on GWG based on serial measurements during pregnancy [18, 19, 28, 30]. Weight changes based on serial measurements using a mixed-effects model approach has been tried [24], but assumed only a linear change in weight throughout pregnancy, which is likely to underestimate the rate of weight gain in the 2nd and 3rd trimester [14]. Another approach is to study the association between smoking status and GWG at discrete time points during pregnancy [18, 19, 28]. Using this approach, however, women who give birth preterm may not contribute to the comparison at the end of the 3rd trimester, despite representing an important subgroup.

The key strength of our study is the longitudinal assessment of GWG, combined with a methodology that is not prone to the bias associated with conventional GWG measures. Our approach facilitates group comparisons at any time point during pregnancy. This feature may contribute to a better understanding of the relative importance of different periods during pregnancy by smoking status. The method allows for variation in the number and timing of measurements between participants, which is favorable in large epidemiological studies.

Limitations of our study include that to in order to explore more complex patterns of GWG beyond a pattern that assumes two distinct rates of gain (i.e., 1st trimester rate of gain and 2nd-3rd trimesters rate of gain), more weight measurements would be necessary for each individual. The effect of smoking cessation on GWG might vary depending on when it occurs (i.e., quitting before or during pregnancy), but we did not have information on exact timing to examine this aspect. Our sample size did not allow us to examine whether pre-pregnancy BMI modifies the effect of smoking on GWG patterns. Future work is warranted on the comparison of GWG patterns by perinatal outcomes.

We identified two distinct high risk groups: women who smoke during pregnancy and gain excessively in the first trimester, and women who quit smoking during pregnancy and have a high rate of weight gain during the 2nd and 3rd trimester. These are well-defined and easily identifiable groups of women who could benefit from targeted prevention strategies to ensure that they gain according to the IOM recommendations. Such interventions are critical given that excessive GWG potentially puts two individuals—the mother and the infant—at risk of overweight, obesity and the associated sequelae later in life [4, 5, 8]. While smoking cessation interventions during pregnancy should continue to be promoted, women who are successful in quitting smoking during this period should be provided with additional support as well as dietary and lifestyle interventions to facilitate appropriate GWG.

## Conclusions

We analyzed GWG patterns based on serial antenatal measurements with an approach (i.e. piecewise mixed-effects models) that is novel in perinatal epidemiology and is not prone to some of the biases associated with conventional GWG measures. We showed that a rapid GWG in the 2nd and 3rd trimester is mostly responsible for the excess total GWG among women who quit smoking, and this new knowledge could allow clinicians to suggest dietary and lifestyle modifications to enable healthy GWG. We uncovered markedly different patterns of GWG among women who never smoked and those who continued smoking during pregnancy that could not have been captured by examining total GWG. These unique trends highlight important time points during pregnancy when specific groups of women could benefit from increased antenatal surveillance and care. Future work involving large epidemiological studies with serial antenatal GWG measurements and analyses that are free of some established biases is needed for a better understanding of factors driving GWG and related perinatal outcomes.

## Additional file

Additional file 1: Figure S1. Histogram of gestational ages in weeks to depict the timing of weight measurements throughout pregnancy. (DOCX 33 kb)

## Abbreviations

ANOVA: Analysis of variance
BMI: Body mass index
CI: Confidence interval
GWG: Gestational weight gain
IOM: Institute of Medicine

## References

[1] Dietz PM, England LJ, Shapiro-Mendoza CK, Tong VT, Farr SL, Callaghan WM (2010). Infant morbidity and mortality attributable to prenatal smoking in the U.S. Am J Prev Med.

[2] Chamberlain C, O’Mara-Eves A, Oliver S, Caird JR, Perlen SM, Eades SJ, Thomas J (2013). Psychosocial interventions for supporting women to stop smoking in pregnancy. Cochrane Database Syst Rev.

[3] Aubin HJ, Farley A, Lycett D, Lahmek P, Aveyard P (2012). Weight gain in smokers after quitting cigarettes: meta-analysis. BMJ.

[4] Gunderson EP, Abrams B (2000). Epidemiology of gestational weight gain and body weight changes after pregnancy. Epidemiol Rev.

[5] Rooney BL, Schauberger CW, Mathiason MA (2005). Impact of perinatal weight change on long-term obesity and obesity-related illnesses. Obstet Gynecol.

[6] Thorsdottir I, Torfadottir JE, Birgisdottir BE, Geirsson RT (2002). Weight gain in women of normal weight before pregnancy: complications in pregnancy or delivery and birth outcome. Obstet Gynecol.

[7] Nohr EA, Vaeth M, Baker JL, Sorensen TI, Olsen J, Rasmussen KM (2008). Combined associations of prepregnancy body mass index and gestational weight gain with the outcome of pregnancy. Am J Clin Nutr.

[8] Yu ZB, Han SP, Zhu GZ, Zhu C, Wang XJ, Cao XG, Guo XR (2011). Birth weight and subsequent risk of obesity: a systematic review and meta-analysis. Obes Rev.

[9] Institute of Medicine and National Research Council of the National Academies (2009). Weight gain during pregnancy: reexamining the guidelines.

[10] Kowal C, Kuk J, Tamim H (2012). Characteristics of weight gain in pregnancy among Canadian women. Matern Child Health J.

[11] Dzakpasu S, Fahey J, Kirby RS, Tough SC, Chalmers B, Heaman MI, Bartholomew S, Biringer A, Darling EK, Lee LS, McDonald SD (2014). Contribution of prepregnancy body mass index and gestational weight gain to caesarean birth in Canada. BMC Pregnancy Childbirth.

[12] McDonald SD, Han Z, Mulla S, Lutsiv O, Lee T, Beyene J (2011). High gestational weight gain and the risk of preterm birth and low birth weight: a systematic review and meta-analysis. J Obstet Gynaecol Can.

[13] Han Z, Lutsiv O, Mulla S, Rosen A, Beyene J, McDonald SD (2011). Low gestational weight gain and the risk of preterm birth and low birthweight: a systematic review and meta-analyses. Acta Obstet Gynecol Scand.

[14] Hutcheon JA, Bodnar LM, Joseph KS, Abrams B, Simhan HN, Platt RW (2012). The bias in current measures of gestational weight gain. Paediatr Perinat Epidemiol.

[15] Hutcheon J, Oken E (2016). Towards defining optimal gestational weight gain. Curr Epidemiol Rep.

[16] Park CK, Krebs L, Lutsiv O, Van BS, Schmidt LA, Beyene J, McDonald SD (2015). Binge eating predicts excess gestational weight gain: a pilot prospective cohort study. J Obstet Gynaecol Can.

[17] Naumova EN, Must A, Laird NM (2001). Tutorial in Biostatistics: Evaluating the impact of ‘critical periods’ in longitudinal studies of growth using piecewise mixed effects models. Int J Epidemiol.

[18] Rode L, Kjaergaard H, Damm P, Ottesen B, Hegaard H (2013). Effect of smoking cessation on gestational and postpartum weight gain and neonatal birth weight. Obstet Gynecol.

[19] Groff JY, Mullen PD, Mongoven M, Burau K (1997). Prenatal weight gain patterns and infant birthweight associated with maternal smoking. Birth.

[20] Washio Y, Higgins ST, Heil SH, Badger GJ, Skelly J, Bernstein IM, Solomon LJ, Higgins TM, Lynch ME, Hanson JD (2011). Examining maternal weight gain during contingency-management treatment for smoking cessation among pregnant women. Drug Alcohol Depend.

[21] Mongoven M, Dolan-Mullen P, Groff JY, Nicol L, Burau K (1996). Weight gain associated with prenatal smoking cessation in white, non-Hispanic women. Am J Obstet Gynecol.

[22] Olson CM, Strawderman MS (2003). Modifiable behavioral factors in a biopsychosocial model predict inadequate and excessive gestational weight gain. J Am Diet Assoc.

[23] Favaretto AL, Duncan BB, Mengue SS, Nucci LB, Barros EF, Kroeff LR, Vigo A, Schmidt MI (2007). Prenatal weight gain following smoking cessation. Eur J Obstet Gynecol Reprod Biol.

[24] Rodrigues PL, de Oliveira LC, Brito AS, Kac G (2010). Determinant factors of insufficient and excessive gestational weight gain and maternal-child adverse outcomes. Nutrition.

[25] Adegboye AR, Rossner S, Neovius M, Lourenco PM, Linne Y (2010). Relationships between prenatal smoking cessation, gestational weight gain and maternal lifestyle characteristics. Women Birth.

[26] Levine MD, Cheng Y, Cluss PA, Marcus MD, Kalarchian MA (2013). Prenatal smoking cessation intervention and gestational weight gain. Womens Health Issues.

[27] Restall A, Taylor RS, Thompson JM, Flower D, Dekker GA, Kenny LC, Poston L, McCowan LM (2014). Risk factors for excessive gestational weight gain in a healthy, nulliparous cohort. J Obes.

[28] Abrams B, Carmichael S, Selvin S (1995). Factors associated with the pattern of maternal weight gain during pregnancy. Obstet Gynecol.

[29] Karachaliou M, Georgiou V, Roumeliotaki T, Chalkiadaki G, Daraki V, Koinaki S, Dermitzaki E, Sarri K, Vassilaki M, Kogevinas M, Oken E, Chatzi L (2015). Association of trimester-specific gestational weight gain with fetal growth, offspring obesity, and cardiometabolic traits in early childhood. Am J Obstet Gynecol.

[30] Rodrigues PL, Lacerda EM, Schlussel MM, Spyrides MH, Kac G (2008). Determinants of weight gain in pregnant women attending a public prenatal care facility in Rio de Janeiro, Brazil: a prospective study, 2005-2007. Cad Saude Publica.

