# Supplementary material for: Are women who quit smoking at high risk of excess weight gain throughout pregnancy?
Source: BMC Pregnancy Childbirth. 2016 Sep 6;16(1):263. doi: 10.1186/s12884-016-1056-z (PMC5011923; doi:10.1186/s12884-016-1056-z)

**Figure S1** Histogram of gestational ages in weeks to depict the timing of weight measurements throughout pregnancy


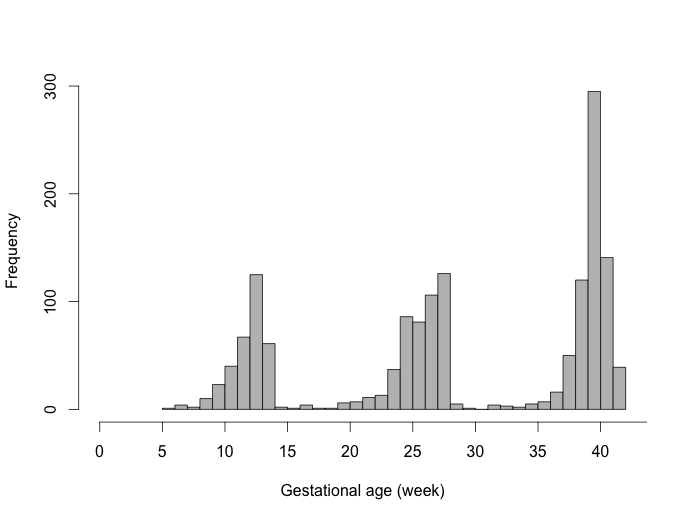

Supplement: Additional file 1: Figure S1. — Histogram of gestational ages in weeks to depict the timing of weight measurements throughout pregnancy. (DOCX 33 kb) [file 12884_2016_1056_MOESM1_ESM.docx]
